# A qualitative study of the perspectives of older people in remote Scotland on accessibility to healthcare, medicines and medicines-taking

**DOI:** 10.1007/s11096-018-0684-y

**Published:** 2018-07-09

**Authors:** Derek Stewart, Kathrine Gibson-Smith, Scott Cunningham, Sharon Pfleger, Gordon Rushworth

**Affiliations:** 1 School of Pharmacy and Life Sciences, Robert Gordon University, The Sir Ian Wood Building, Garthdee Road, Aberdeen, AB10 7GJ UK; 2 NHS Highland, Assynt House, Beechwood Park, Inverness, IV2 3BW UK; 3 Centre for Health Science, Highland Pharmacy Education and Research Centre, Old Perth Road, Inverness, IV2 3JH UK

**Keywords:** Access to healthcare, Convenience, Interview study, Rural health services, Scotland

## Abstract

*Background* Recent evidence highlights the disproportionate rates of health inequality often experienced within remote and rural communities. Access to medicines within remote and rural communities may also prove problematic. *Objective* The aim was to understand the perspectives of older people in the most remote areas of the Scottish Highlands on issues of accessibility to healthcare, medicines and medicines-taking. *Method* Qualitative, semi-structured one-to-one interviews with 13 residents aged 65 years and over in the most remote and rural areas of the Scottish Highlands. Interviews were audio-recorded, transcribed and analysed using a framework approach. *Setting* Remote and rural areas in the Scottish Highlands. Participants had previously completed a cross-sectional survey. *Main outcome measure* Themes surrounding the perspectives of older people in the most remote areas of the Scottish Highlands on issues of accessibility to healthcare, medicines and medicines-taking. *Results* Healthcare was considered convenient, and positive relationships with providers being important. Review of medicines was perceived to be the remit of the doctor, with pharmacists seen as valuable suppliers of medicines. *Conclusion* Based on this qualitative study, experiences of access to healthcare, including community pharmacy, medicines and medicines-taking within this sample of older adults resident in the most remote areas of the Scottish Highlands are widely variable. There may be an unmet educational need, amongst residents, with regard to awareness of the role of pharmacists, the services they can provide and the benefits which may be experienced as a consequence of engagement.

**Electronic supplementary material:**

The online version of this article (10.1007/s11096-018-0684-y) contains supplementary material, which is available to authorized users.

## Impacts on practice

- In remote and rural areas of Scotland, positive relationships with health professionals are highly valued by older people and are more important than convenience of services.
- While pharmacists are valued as suppliers of medicines for older people, there is a need to raise awareness of other aspects of pharmaceutical care and likely benefits.
- Older people in remote and rural areas are highly dependent on others for support hence pharmacists should be alert to issues around medicines supply and adherence.

## Introduction

In Scotland, rural communities are defined in terms of their settlement size and may include ‘accessible rural’ (with a population of less than 3000 people, and within a 30 min drive time of a settlement of 10,000 or more) or ‘remote rural’ areas (with a population of less than 3000 people, and with a drive time of over 30 min to a settlement of 10,000 or more) [1]. There are well-recognised issues of access to healthcare and supporting population engagement with healthcare in remote and rural communities [2]. These issues are compounded by the difficulties in securing an appropriately qualified healthcare workforce [3]. Recent evidence derived from 174 countries has highlighted the disproportionate rates of health inequality often experienced within remote and rural communities [4]. For example, rural areas often experience higher rates of suicide, alcohol related disease, accidents and palliative care treatment [5].

Numerous studies have identified a range of difficulties commonly encountered in relation to healthcare in remote and rural communities: problems accessing care; centralised healthcare services; distance; travel costs; waiting times; service hours; relatability of the GP; starting the care process again; utilisation of emergency services; health deterioration; and making trade-offs between safety and accessing healthcare [6–13].

Access to medicines within remote and rural communities may also prove problematic. A systematic review of literature on rural patients living with long term conditions reported a demand within those living in remote and rural communities for access to pharmacists [7].

NHS Highland is the largest geographical health board area in the United Kingdom (UK), covering 41% of the land mass of Scotland, but with a population representing only 6% of Scotland whereby 40% of whom live in ‘remote rural’ locations [1]. The Scottish Government 2017 ‘Achieving Excellence in Pharmaceutical Care’ strategy for Scotland outlines a series of commitments from the Scottish Government with regard to the provision of pharmaceutical care which “…focuses the knowledge, responsibilities and skills of the pharmacist on the provision of drug therapy with the goal of achieving definite therapeutic outcomes toward patient health and quality of life.” Key commitments of the strategy relate to enhancing access to pharmaceutical care in remote and rural communities and the provision of improved pharmaceutical care for people being cared for in their own homes [14]. Similarly, the ‘Delivering for Remote and Rural Healthcare’ report of the Scottish Government highlights the importance of building community resilience within remote and rural areas whereby communities utilise health services which are available to them [5].

Recent research conducted in Scotland explored access to medicines and healthcare services within remote and rural areas. A large-scale survey of residents (n = 2913) aged ≥ 18 years in rural Scotland highlighted that whilst access to medicines was typically considered convenient, there were inherent challenges for older adults (≥ 80 years) and those living alone [15]. A later survey conducted in the same geographical area amongst those aged ≥ 60 years (n = 1042) reported that older age respondents were significantly more likely to state that their access to healthcare services was not convenient and that those in the most rural areas had issues around access to pharmacies [16].

The Scottish Government places much focus on the care of older people, publishing ‘Reshaping Care for Older People’ which articulates the goal to ensure that ‘older people are valued as an asset, their voices are heard and they are supported to enjoy full and positive lives in their own home or in a homely setting’ [17]. Several government strategies place emphasis on a health and social care shift towards ‘personalisation’, whereby people become more involved in how services are designed and receive the support that is most suited to them and where older people are supported to live a healthy life whereby long terms conditions are managed [17–20]. Further, The Scottish Government ‘Better Health, Better Care Action Plan’ stipulates the importance of there being community based services which promote the delivery of care at home or within the community. The document also highlights the role of community pharmacies in the provision of accessible services [21].

### Aim of the study

Given the policy direction of the Scottish Government and the findings of several survey based studies, there is a need for qualitative research which focuses on older people living in the most remote areas.

The aim of this research was understand the perspectives of older people in the most remote areas of the Scottish Highlands on issues of accessibility to healthcare, medicines and medicines-taking.

### Ethics approval

This study was approved by the Ethical Review Panel of the School of Pharmacy and Life Sciences at Robert Gordon University, UK; the North of Scotland Research Ethics Committee advised that the study was exempt from NHS ethical review.

## Methods

### Design

The design was a qualitative methodology of semi-structured, one-to-one interviews.

### Sampling frame and sampling

The sampling frame comprised members of the general public aged ≥ 60 years resident in very remote rural areas of the Scottish Highlands, as defined by the Scottish Government classification (areas with a population of < 3000 and with a drive time of over 60 min to a settlement of ≥ 10,000) [1]. These individuals had participated in a cross-sectional survey on access to general practitioners, community pharmacies and prescribed medicines [16], and had expressed interest in being involved in further research: 133 were interested and of these, there were 50 individuals taking 5 or more medicines. A purposive sampling approach was used within the interviews in an effort to obtain the perspectives of those prescribed most medicines.

### Interview schedule development

A semi-structured interview schedule was developed to explore access to general practice and community pharmacy, medicines and medicines taking practices. The schedule was reviewed for credibility by individuals with extensive expertise in policy, practice and research related to older people and medicines: two senior clinical pharmacists; two academic researchers; a senior pharmacist based in the Scottish Government; and a consultant physician specialising in the care of older people. Following minor revision, the schedule was piloted with two survey respondents resident in less remote areas.

### Recruitment

Potential participants were mailed a study invitation letter, information leaflet and consent form. If interested, they were requested to complete and return the consent form, providing a contact telephone number and details of suitable days and times to be contacted to arrange an interview.

### Data generation

Interviews of approximately 20 min duration were conducted by trained researchers. The interviews were digitally audio-recorded, transcribed verbatim and checked for transcribing accuracy prior to analysis. Data saturation was established using the approach of Francis et al. [22] with an initial sample size of ten and a stopping criterion of three i.e. no new themes emerged from three consecutive interviews.

### Data analysis

Members of the research team met to agree the initial coding framework. Transcripts were analysed independently by two researchers using the Framework Approach of: data familiarisation; identifying constructs; indexing; charting; mapping; and interpreting [23].

## Results

Data saturation was considered to have occurred following 13 interviews. Four overarching themes were identified from the analysis. Subthemes are presented in accordance with each overarching theme.

### Accessible healthcare

#### Healthcare that is accessible and convenient

Almost all interviewees considered their GP practices to be both accessible and convenient, irrespective of the mode of travel and distance to be travelled. Whilst some accessed by walking or cycling, others required transportation. Mode of transportation was typically a car, however one interviewee reported that the only means of accessing their local GP practice was via ferry.

We live on a small island which is 25-30 minute ferry journey to the [location]; that runs from about 8 o’clock until about 6 o’clock at night, after that there is no service…if we have to go visit the doctor in [location], we have got again a 30 minute ferry ride and then another 25 minute car journey [Interviewee 9].

The two interviewees who were located a long distance from a GP practice highlighted that a healthcare professionals from their local practice visited patients on a weekly basis. However, one interviewee noted that such visits were dependent on the weather conditions since they may impede on accessibility.

And once a week – again weather permitting in the winter – we have a doctor who comes over and visits from the [name] practice

[Interviewee 9].

Interviewees also reported that pharmacies were accessible, often located close to the GP practice or within the GP practice itself.

It’s in the same town, 36 miles away, again

[Interviewee 11].

#### Dependence on others

Some interviewees depended on others for transport, for example, family members or a local car scheme, run by volunteers.

There is a car scheme which runs, you know, from volunteers and we can organise that the day before – and if need be on the same day in an emergency

[Interviewee 10].

It was also highlighted, by one interviewee, that whilst their GP practice was accessible this was contingent on having access to transport.

Well it is pretty convenient in that I can see some doctor when I want, whenever I want; as long as I have transport

[Interviewee 10].

Whilst one interviewee noted that they walked to the pharmacy, for others, transportation was required. As prior, one interviewee highlighted that someone needed to take them to the pharmacy and another, noted that they could only drive to the pharmacy since there was no bus service operating.

Yes [drives to pharmacy] because there is no bus I couldn’t get about any other way

[Interviewee 3].

#### Being organised is essential

Many commented that while access was not an issue, they also described the need to be organised when visiting the GP or pharmacy to get the most out of one visit. This often involved collecting medicines or errands for others.

If you have to go – if people have to go over for medicines or anything; it is a half day journey really. And what you try to do is organise it so you do your weekly shopping as well, and get anything else there because we have a small shop on the island but it is just the basics so anything…we…you try to make sure you have got more than one reason to go over because it is pretty expensive. And we try to make everything…the other thing is we tend to get organised with other people so we pick up several peoples’ prescriptions at the same time

[Interviewee 9].

### Variation in satisfaction with healthcare irrespective of location

#### Importance of positive relationships

Interviewees were generally satisfied with their healthcare in terms of both accessibility and the service received. The importance of developing close relationships with healthcare professionals and support staff was emphasised.

…well it is rather difficult to say. But–you know–we do…we do get a good service from them; even being remote. Very often they are on the end of the phone. The receptionists in [location] they usually understand our position and if we can’t speak to a doctor they regularly will get the doctor to phone back. Again we have a good relationship with them individually, yes

[Interviewee 9].

This same individual, in a very remote location, commented that their health professionals almost knew everyone personally.

To get a decent practice, you have to have a number of people and actually we have a very good practice in [location] and the doctors…well majority of the practice know us and know most of the individuals on the island almost personally. Which you know, is excellent

[Interviewee 9].

Several interviewees who were not satisfied with aspects of their healthcare cited issues of lack of healthcare professional cover in their community with consequent use of locums who were less familiar with their background and difficulties in communication.

I seem to think we have a lot of locums at the moment and my doctor is off on maternity leave so and she has had two babies in two years so it’s been a bit awkward. That way to have had locum doctors and you know it’s hard to speak to them because they don’t know your family background

[Interviewee 6].

### Reviews of medicines

#### Role of health professionals in reviews of medicines

While interviewees acknowledged the role of the GP in reviewing their medicines, the contribution of the pharmacist was less recognised. The frequency of review by the GP varied from an annual review to being reviewed during each consultation.

Our practice is very good; they do give us a once a year review of our medicines

[Interviewee 8].

The doctor does [discuss all medicines] when I got and see him. He will say well yes you should be kept carrying on with these medicines as previously prescribed. So yes, I discuss it with the doctor when I see him

[Interviewee 9].

Interviewees generally perceived that medicines review was more the remit of the doctor than the pharmacist.

Oh I never speak to the pharmacist about it. Just the doctor

[Interviewee 4].

This interviewee did, however, comment on the very positive input of the pharmacists to their care, particularly around the use of over-the-counter medicines.

… I think the pharmacist would be very helpful and supportive and suggest something that I might be able to get without using a prescription or they would advise me to go and see, you know, see the doctor. Whenever I have discussed, in the chemist, or in the one attached to [the GP surgery] over the road, they’ve been very, you know, helpful and supportive. But I haven’t actually used them very much

[Interviewee 4].

Others also described situations where the pharmacist had intervened over their prescription medicines.

I can tell you exactly, because I had a flare up of arthritis, and I asked for some ibuprofen, because the doctor had suggested I could use that as a top up and when my son went to collect them, they telephoned me and said did I know that I shouldn’t take ibuprofen because I’d had an ulcer a while back and eh, I had to explain that the doctor had prescribed omeprazole to counteract the side effects of the ibuprofen

[Interviewee 2].

However, it was noted by some, that they did not feel that discussing their medicines with pharmacy staff would be beneficial,

No I’ve got all the help I need from the doctor

[Interviewee 5].

and others had no experience of the pharmacist reviewing their medicines.

They don’t discuss it, no

[Interviewee 1].

Oh not for a long time [last time pharmacy staff discussed medicines on prescription]

[Interviewee 11].

### Autonomy and competence

#### Role of routine in managing medicines

Interviewees considered themselves to be autonomous and competent regarding managing their medicines themselves describing how they often ordered, collected and organised their medicines themselves, with some reporting that they filled their own compliance aids with prescription medicines. It was also highlighted that ensuring that medicines were well managed and organised was particularly important when living in remote locations since the weather may impede on any plans.

You certainly have to plan ahead! You certainly have to be more prepared; I mean not only medically but food-wise and stuff, so you do tend to have to plan ahead, for anything that you do. And with the winter you also have to have one eye on the weather; whether the ferry will run or not

[Interviewee 9].

Interviewees did not report any difficulties in taking or managing their medicines and one emphasised, that if they or their partner experienced any difficulties, the other would help. Interviewees generally recognised the importance of taking their medicines and were aware of the negative consequences of not adhering to their treatment regimen.

Well I’d probably be dead if I didn’t have them so I think it’s very important

[Interviewee 4].

Whilst one interviewee was unable to collect their own medicines, they felt that it was their own responsibility to manage medicines.

I think they do far too much for people nowadays. It makes you give up

[Interviewee 2].

Taking responsibility for managing medicines was echoed by a number of interviewees however, two interviewees highlighted that it was their responsibility to do so as long as they were capable.

Well I think it’s my responsibility so long as I’m mentally able

[Interviewee 4].

Although the responsibility, to manage medicines, was perceived to be that of the patient by a number of interviewees, it was also noted by some, that they did not know what may happen if they did not take their medicines, and were following the advice of their GP.

I have no idea really I haven’t tried not having them to see if anything happens so I am assuming it is important because the doctor assured me that it was necessary so I take his word for it

[Interviewee 5].

Two interviewees reported that they had made the decision to stop taking some of their medicines, since they either made them feel worse or due to experience of negative side effects.

Honestly, yes. I hate the effect that iron tablets have on me. I would rather eat more sensibly. It’s because I have low iron, you know, low haemoglobin. So they prescribed iron tablets and I loathe them. Well its constipation, and I’d rather not get constipated I’d rather eat spinach

[Interviewee 2].

#### Role of others in managing medicines

Interviewees largely did not report discussing their medicines with others although some did discuss their medicines with family members, particularly their siblings, children or those with experience as a healthcare professional.

I do speak to my sisters a lot. I think my family are of the type who use medicines as little medicine as possible. That’s the type they are. But yes, I speak to my sisters a lot

[Interviewee 10].

Whilst, some interviewees were dependent on others, to a degree, to manage their medicines particularly in relation to collecting and organising medicines to be taken, others who were managing their medicines themselves, described that they may need to rely on others to help if they were no longer able. This reliance extended to healthcare professionals, family and neighbours. It was highlighted, that within remote communities, some may require assistance from others and are often dependent on neighbours to assist them in managing medicines.

…there are people who have serious problems with living alone and no transport and depend on neighbours in isolated areas

[Interviewee 1].

#### Strategies around medicines adherence

A number of interviewees reported that they always remembered to take their medicines, this was enhanced by their routine and also, one interviewee highlighted that physical prompts reminded them to take their medicine.

I leave it on the kitchen table to try not to forget

[Interviewee 1].

Whilst a number of interviewees did not forget to take their medicines, some reported that they did, on occasion, forget to take their medicines.

Oh about a couple of times a year I might just forget but I just leave that out and go onto the next one so I would say very, very seldom do I forget

[Interviewee 4].

## Discussion

Four key themes emerged in these qualitative interviews with older adults in some of the most remote and rural areas of Scotland. Healthcare was considered to be convenient irrespective of the location or mode of travel required, with the importance of dependence on others and being well-organised being highlighted. While there was variation in the levels of satisfaction with healthcare services, positive relationships with healthcare providers were important and could be disrupted by regular changes in personnel or difficulties in staff recruitment. Review of medicines was perceived to be the remit of the doctor, with pharmacists seen as valuable suppliers of medicines. Medicine taking was not perceived to be an issue, interviewees considered themselves to be competent and highly organised, although often required the support of others.

## Strengths and limitations

This qualitative interview study adds to the limited body of knowledge on issues of access to healthcare, medicines and medicines-taking for those resident in the most remote areas. Research trustworthiness was assured via utilisation of a number of strategies to promote credibility, transferability, dependability and confirmability. These included: integration of widely used research methods (credibility); expert review of data generation materials (credibility); training of interviewers (credibility); providing accurate in-depth descriptions of the research setting, procedures and study participants (transferability/dependability/confirmability). A further strength, was the application of recognised saturation principles to the data to ensure data saturation had occurred [22]. The study findings are however, limited to the participants sampled and hence, those agreeing to be interviewed may not have been representative of all older people residing in remote areas. An additional limitation of the study may be that participants repeated a response which was similar to the manner in which the question was asked, an issue which may be inherent in semi-structured interviews due to having pre-defined interview schedules. However, interviewers adopted additional probing in an effort to overcome potential issues with repetition.

## Interpretation

All interviewees reported access to healthcare to be both accessible and convenient. Whilst it was reported that access to healthcare was some distance away, perhaps requiring transport via ferry, this was not necessarily perceived negatively. The only difficulties reported in relation to access were due to adverse weather conditions. This finding corresponds with existing mixed methods research conducted amongst urban and rural residents in Scotland. Indeed, Farmer et al. [24] reported that rural residents had higher levels of satisfaction than those living in urban areas regarding their access to health services. Furthermore, the finding that interviewees either currently, or would in the future, rely on others to assist them in gaining access to healthcare has also been reported by others [10]. Such ‘community resilience’, defined as “a collective and collaborative response within communities to promote independence”, is often a feature of remote areas. The Scottish Remote and Rural Steering Group report notes positive consequences for those communities enabled to care for themselves, employ adequate resources, engage in self-care and to rely on volunteers within the community [5]. It has been advocated that individual expression, within capabilities, of both autonomy and competence may further promote resilience [25].

The importance of organising medicines and creating routines were regarded as key to autonomy and competence. These results may be viewed via the lens of Self Determination Theory (SDT) which posits three tenets critical to fulfilling an individuals’ psychological needs: autonomy (the requirement to feel a sense of responsibility for executing a behaviour); competence (the need for individuals to feel effective in interactions with their environment); and relatedness (to feel a sense of belongingness and connectedness with others) [26, 27]. The discussions around autonomy and competence, in relation to medicines-taking, within this sample perhaps highlights the importance to older adults of maintaining both autonomy and competence in accordance with their degree of capability.

Familiarity with GPs was regarded as an important factor when considering satisfaction with healthcare and it was perceived that it was often difficult to build a rapport where temporary staff such as GP locums were used. Evidence from the Primary Care Workforce Survey suggests that the use of locum GPs has markedly increased in recent years, largely as a result of growing GP vacancy rates [28]. Recruitment and retention of GPs in remote and rural practices remains a significant challenge in Scotland [3] and other remote areas globally [29].

Given the particular difficulties in recruiting the medical workforce to remote and rural areas generally, there is a need to explore models of healthcare which can be delivered by other healthcare professionals. The high prevalence of multimorbidity in older people is associated with prescribing of multiple medicines. Prescribing data for Scotland highlight that almost 30% of those aged 60–69 years receive four to nine medicines and 7.4% ten or more medicines; in those aged ≥ 80 years the figures are even higher at 51.8 and 18.6%, respectively [30]. Scottish Polypharmacy Guidance on optimising medicines outlines the importance of healthcare professionals, particularly pharmacists, in participating in medication reviews [31]. The Scottish Government ‘Achieving Excellence in Pharmaceutical Care in Scotland’ strategy advises a vision for pharmacy ‘as an integral and enhanced part of a modern NHS in Scotland’. The vision suggests that pharmacists and pharmacy technicians’ working in remote and rural communities will create greater access to pharmaceutical care, enable achievement of better health outcomes and assist in sustaining services. Further, the strategy places community pharmacy at the hub of care advising that there should be increased access as a first port of call for the management of self-limiting illnesses and in providing support for self-management of long term conditions [14].

Interviewees however reported that medicines reviews were generally conducted by GPs, with very few reporting any pharmacist involvement, or indeed any indication that pharmacist involvement was warranted. This finding is consistent with recent survey based research in this geographical area, with a minority of respondents reporting regular pharmacist review of medicines for chronic conditions [16]. Taken together, the findings of these studies suggest that there is a lack of perceived need amongst remote and rural residents in Scotland for the provision of pharmaceutical care and pharmacist input in healthcare. This is perhaps surprising given the current policy directions in Scotland which highlight the potential role for pharmacists in being increasingly involved in person–centred holistic patient care [14]. Further, the recently published General Medical Services Contract in Scotland envisions pharmacists delivering key pharmacotherapy services within GP practices including specialist polypharmacy reviews and specialist clinics. Such models are in development, particularly within the GP practice setting and have demonstrated positive benefits within the Scottish Highlands [32].

There may also be an unmet educational need, amongst older residents, with regard to awareness of the role of pharmacists, the services they can provide and the benefits which may be experienced as a consequence of engagement. Increasing awareness may somewhat benefit service utilisation since many patients seem to be unaware of the services which are provided by pharmacies and pharmacy teams. Moreover, this may also be an important avenue to pursue in that it may assist in building trust and confidence between members of the public and pharmacists and the services they can provide. Further, the findings also suggested that pharmacists did not proactively discuss medications with customers and whilst there may be a myriad of reasons for not doing so, it could likely be due to timing and workload issues. Such discussions may promote trust between pharmacists and the community, and should hence, be encouraged.

Hence, given the findings of the research there is a requirement for further research to explore awareness of the availability of community pharmacy services and perceptions of pharmacy within remote and rural communities. In addition, it may be beneficial to explore patients’ acceptability of new services and also what would be considered appropriate means of delivery whether this be face-to-face or digital.

## Conclusions

Based on this qualitative study and within the sample studied, experiences of access to healthcare, including community pharmacy, medicines and medicines-taking within older adults resident in the most remote areas of the Scottish Highlands are widely variable. There may be an unmet educational need, amongst residents, with regard to awareness of the role of pharmacists, the services they can provide and the benefits which may be experienced as a consequence of engagement. There is a need for quantitative research to test the generalisability of these issues. In addition, further in-depth research which seeks to explore awareness of the availability of pharmacy services and perceptions of pharmacy within remote and rural communities is warranted.

## Electronic supplementary material

Below is the link to the electronic supplementary material.

Supplementary material 1 (DOCX 411 kb)
