# Supplementary material for: A qualitative study of the perspectives of older people in remote Scotland on accessibility to healthcare, medicines and medicines-taking
Source: Int J Clin Pharm. 2018 Jul 9;40(5):1300–8. doi: 10.1007/s11096-018-0684-y (PMC6208609; doi:10.1007/s11096-018-0684-y)
Supplement: Supplementary file 1 — Supplementary material 1 (DOCX 411 kb) [file 11096_2018_684_MOESM1_ESM.docx]

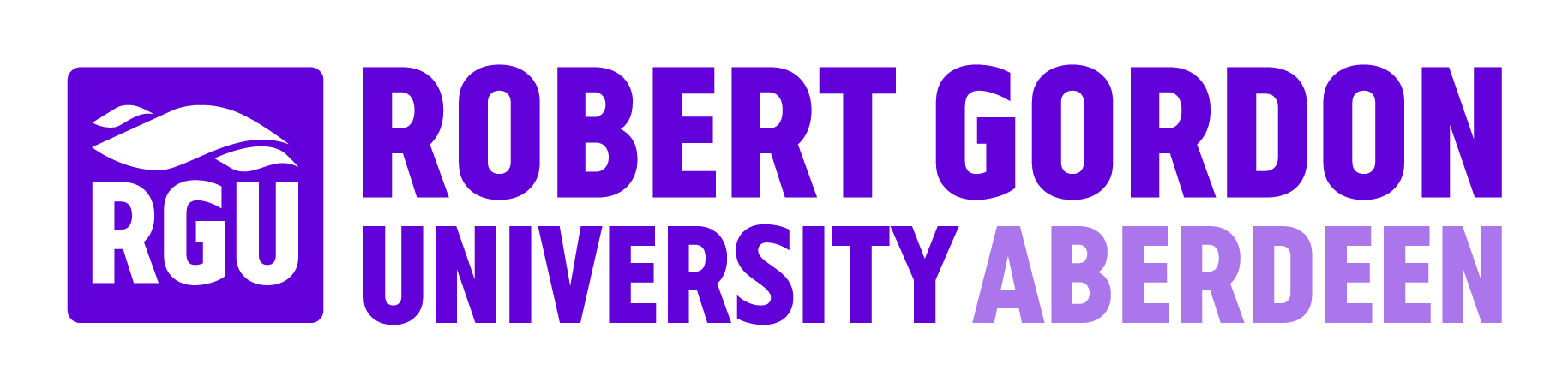

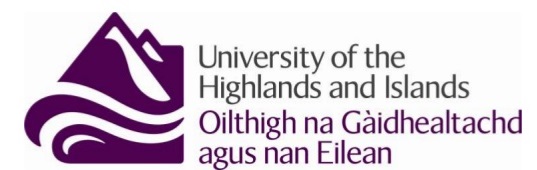


**Research team**

Professor Derek Stewart Mr Gordon Rushworth Dr Sarah-Anne Munoz

Dr Scott Cunningham Mrs Sharon Pfleger Dr Jenny Hall

**A qualitative study of the behaviours and experiences of older people living within the community setting of the Scottish Highlands in relation to pharmaceutical care services**

**Research aim**

To describe and understand the behaviours and experiences of older people living within the community setting of the Scottish Highlands in relation to pharmaceutical care services.

For the purposes of this research, pharmaceutical care services are defined as the supply, support, advice and review of prescription medicines.

**Research design and method**

Qualitative, phenomenological, interpretative study of semi-structured telephone interviews. Interviewees will be sampled from those older people responding to a cross-sectional survey study of pharmaceutical care services in the Scottish Highlands who agreed to participate in further research.

There will be two groups of interviewees, with data generation continuing to the point of saturation in each group to allow comparisons of themes to be drawn. The groups will be

a. those with no issues of convenience to services, no worries or barriers identified from survey responses

b. those with issues

This research will gain more depth on areas and issues identified in the questionnaire.

The students will be trained in qualitative interviewing, how to probe for further information etc. and will audio-record the telephone interviews.

**The specific objectives in relation to these older people are to:**

1. identify issues around pharmaceutical care services by critically reviewing published literature

2. describe and understand behaviours and experiences in relation to access to pharmaceutical care services

3. explore any facilitators of and barriers to pharmaceutical care services

4. to compare responses in the two groups

5. describe and understand concerns and potential solutions

**Draft interview schedule**

We have questionnaire data for each individual, although this may have changed and could be confirmed at the start of the interview

**Suggested questions for comment**

**Section 1, experiences**

**1. Tell me about how convenient you think your GP practice is for you?**

*What does convenience mean to you?*

*Is it an issue for you? Please tell me more*

*Why do you think this?*

*Could it be more convenient and how?*

**2. Tell me about how convenient you think your pharmacy (chemist) is**

*What does convenience mean to you?*

*Is it an issue for you? Please tell me more. What do you do?*

*Why do you think this?*

*Could it be more convenient and how?*

**3. Please tell me how you usually get your medicines you get on prescription**

*Include ordering the prescription, collecting the prescription, collecting the medicines, how often*

*Is it convenient for you?*

*What does convenience mean to you?*

*Is it an issue for you? Please tell me more. What do you do?*

*Why do you think this?*

*Could it be more convenient and how?*

**4. How does this fit into the things you do every day?**

*Are there any issues and what are they? – tell me more*

*How could this be made easier for you?*

**5. Tell me about when your doctor or anyone else in your GP practice last talked to you about all your medicines you get on prescription to see that they are still working and that you still need them**

*Do you remember who spoke to you and what was discussed?*

*Was this for all medicines or just some of them*

*Do you think your medicines are still working for you and that you still need them?*

*Could your doctor or anyone else in your GP practice do more to help you?*

**5. Tell me about when your pharmacist (chemist) last talked to you about all your medicines you get on prescription to see that they are still working and that you still need them**

*Where did this take place?*

*Do you remember what was discussed?*

*Was this for all medicines or just some of them?*

*Could your pharmacist (chemist) do more to help you with your medicines?*

*Tell me more about this*

*Could your pharmacist (chemist) do more to help you with your health in general?*

*Tell me more about this*

**6. Do you ever speak to your family or friends about your medicines you get on prescription?**

*Tell me about this*

**7. Tell me about how you take your medicines you get on prescription and remembering to take or use them**

*Do you have any issues – tell me?*

*What might help you to make it easier?*

**Section 2 is about behaviours**

***Information for reviewers***

The theoretical domains framework is the theoretical lens for the qualitative interviews around behavioural determinants. There are 14 domains as shown below; not all of the domains are relevant for the interviews.

| **TDF Domains** | **Description** |
| --- | --- |
| Knowledge | An awareness of the existence of something |
| Skills | An ability or proficiency acquired through practice |
| Social/Professional Role and Identity | A coherent set of behaviours and displayed personal qualities of an individual in a social or work setting |
| Beliefs about Capabilities | Acceptance of the truth, reality, or validity about an ability, talent, or facility that a person can put to constructive use |
| Optimism | The confidence that things will happen for the best or that desired goals will be attained |
| Beliefs about Consequences | Acceptance of the truth, reality, or validity about outcomes of a behaviour in a given situation |
| Reinforcement | Increasing the probability of a response by arranging a dependent relationship, or contingency, between the response and a given stimulus |
| Intentions | A conscious decision to perform a behaviour or a resolve to act in a certain way |
| Goals | Mental representations of outcomes or end states that an individual wants to achieve |
| Memory, Attention and Decision Processes | The ability to retain information, focus selectively on aspects of the environment and choose between two or more alternatives |
| Environmental Context and Resources | Any circumstance of a person's situation or environment that discourages or encourages the development of skills and abilities, independence, social competence, and adaptive behaviour |
| Social Influences | Those interpersonal processes that can cause individuals to change their thoughts, feelings, or behaviours |
| Emotion | A complex reaction pattern, involving experiential, behavioural, and physiological elements, by which the individual attempts to deal with a personally significant matter or event |
| Behavioural Regulation | Anything aimed at managing or changing objectively observed or measured actions |

Some of these will be covered earlier. The order of the questions here is in relation to the domains but will be sorted later to flow easier.

**Knowledge**

**1. If you needed it, where would you get help or support with getting your medicines you get on prescription?**

*Why would that be your first point for support? Tell me more*

*Have you actually asked for help or support, say in the last 6 months?*

*Did you get the help or support that you needed?*

***2*. If you needed it, where would you get help or support with taking or using your medicines you get on prescription?**

*Why? Tell me more*

*Have you actually asked for help or support, say in the last 6 months?*

*Did you get the help or support that you needed?*

**Skills – covered under beliefs of capabilities**

**Social/professional role and identity**

**3. Do you think it is yours or someone else’s responsibility to make sure that you get the medicines that you need?**

*Why? Tell me more*

*If someone else, who and why?*

**Beliefs about Capabilities**

**4. Do you have any problems of difficulties getting your medicines you get on prescription?**

*Tell me more about that - why is it difficult, for how long has this been an issue?*

*What do you do when this happens?*

*What might make it easier for you?*

**5. Do you have any problems of difficulties taking or using your medicines you get on prescription?**

*Tell me more about that - why is it difficult, for how long has this been an issue?*

*What do you do when this happens?*

*What might make it easier for you?*

**Beliefs about Consequences**

**6. How important is it to you to get your medicines you get on prescription?**

*Tell me more about that – why is it so important to you?*

*What would happen if you didn’t?*

*Are any of your medicines more important than others?*

**7. How important is it to you to take or use your medicines you get on prescription?**

*Tell me more about that – why is it so important to you?*

*What would happen if you didn’t?*

*Are any of your medicines more important than others?*

**Memory, Attention and Decision Processes**

**8. How do you remember to get your medicines you get on prescription?**

*Do you have a routine or reminder?*

*Do you ever forget to get your medicines?*

*Tell me about this*

**9. Do you ever choose not to get your medicines you get on prescription?**

*Tell me about this*

*What happens?*

*Do you worry about it?*

**10. How do you remember to take or use your medicines you get on prescription?**

*Do you have a routine or reminder?*

*Do you ever forget to take your medicines?*

*What happens?*

*Do you tell anyone? Who? What do they say?*

**11. A lot of people find it hard to take a lot of medicines. Do you ever choose not to take or use your medicines you get on prescription?**

*Tell me about this*

*What happens?*

*Do you worry about it?*

**Environmental Context and Resources (much covered under experiences, convenience)**

**12. Do you have or need any help in getting your medicines you get on prescription?**

*Tell me about this - who, how etc*

*Could anything or anyone else help – tell me?*

**13. Do you have or need any help in taking your medicines you get on prescription?**

*Tell me about this - who, how etc*

*Could anything or anyone else help – tell me?*

**Social Influences**

**14. Do you ever speak to any of your family or friends about getting or taking your medicines you get on prescription?**

*Tell me about this – who, when, what said*

**15. Could any of your family or friends help you more in getting your medicines you get on prescription?**

*Tell me about this – who, how*

**16. Could any of your family or friends help you more in taking your medicines you get on prescription?**

*Tell me about this – who, how*

**Emotion**

**17. Do you ever worry about getting your medicines you get on prescription either now or in the future?**

*Tell me about this – what are you worried about?*

*How you spoken to anyone about this – who, when, what?*

*Is there anything that could be done to help?*

**18. Do you ever worry about taking your medicines you get on prescription either now or in the future?**

*Tell me about this – what are you worried about?*

*How you spoken to anyone about this – who, when, what?*

*Is there anything that could be done to help?*

**Behavioural Regulation**

**19. In the recent past (say the last 6 months), have you changed anything about getting your medicines you get on prescription?**

*Tell me about this – what, why and what happened?*

**20. In the recent past (say the last 6 months), have you changed anything about taking your medicines you get on prescription?**

*Tell me about this – what, why and what happened?*

**Other comments**

**Is there anything else you would like to add about getting or taking your medicines you get on prescription?**


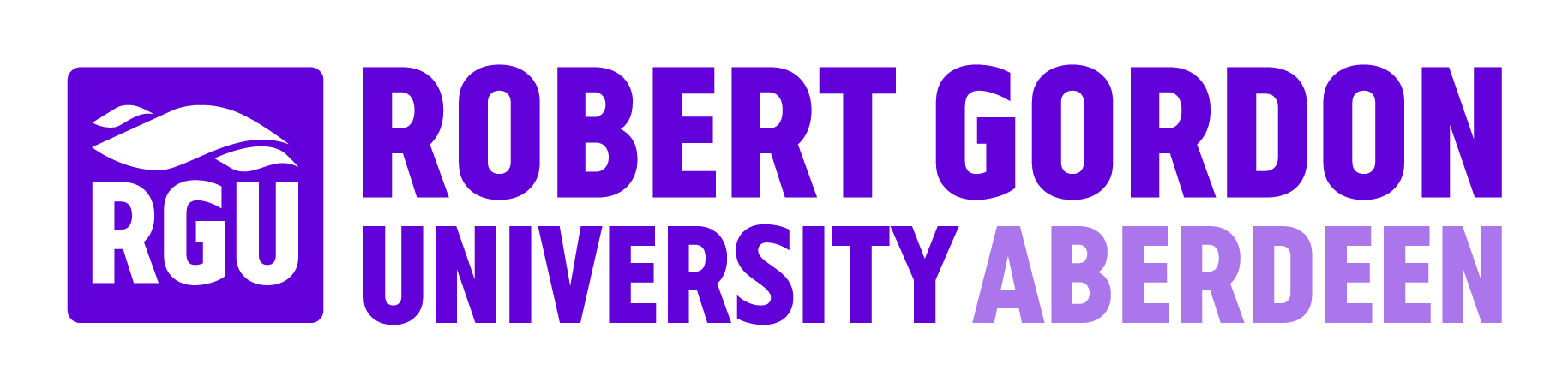

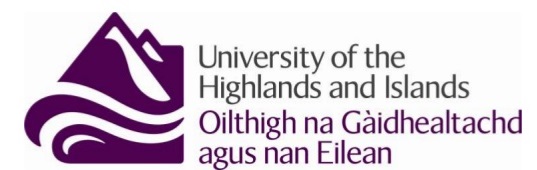


**Research team**

Professor Derek Stewart Mr Gordon Rushworth Dr Sarah-Anne Munoz

Dr Scott Cunningham Mrs Sharon Pfleger Dr Jenny Hall

**A qualitative study of the behaviours and experiences of older people living within the community setting of the Scottish Highlands in relation to pharmaceutical care services**

**SEMI-STRUCTURED INTERVIEW SCHEDULE** for PCI patients

| *****SWITCH ON THE AUDIO RECORDER*****  **Put the phone on ‘Speaker phone’ (loud speaker** 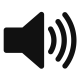 **button at bottom right on the CISCO RGU phones. Check volume is sufficient for recording.**  **Phone the patient: dial ‘9’ for outside line and then the patients contact number.** | **Name of Participant** | **Date** | **Time**  **00:00** |
| --- | --- | --- | --- |

**A. Introduction**

Hello, can I speak to [patient name], please?

| **IF NO:** OK, I had arranged to call at this time. Should I call again in ten minutes or email them to re-schedule? | Write the outcome in your diary chart and take the appropriate action (call back, email) |
| --- | --- |

Hello, [patient name]. I’m [student’s name], the pharmacy student from Robert Gordon University ringing / visiting to interview you about how you use medicines. Are you still okay to do the interview now? It will take around ten minutes.

| **IF NO:** That’s okay. When would you like me to call back?  (offer to email if pharmacist is not sure)  Thanks [name of pharmacist]. I’ll call again on day/date/time. Bye. | Write the new day/date/time here and in diary chart: |
| --- | --- |

**IF YES continue:** That’s great, thank you.

**B. Housekeeping**

As you are aware from the information sheet and consent form, this conversation is being audio recorded to make sure that I don’t miss important points by relying on my memory or notes but I would emphasise that it is confidential. Are you still OK with that?

| **IF NO:**  That’s fine. I won’t use the audio recorder but I’ll need a bit more time to write down notes as we go through the sections and I may ask you to repeat some answers. | Reminders:   - Make sure the audio recording is activated - Take time to write detailed notes - If in doubt, ask the pharmacist for clarification before you move on to the next section |
| --- | --- |

If you decide after the interview you no longer wish to be a part of the research, please let us know within the next seven days. The contact details are on the information sheet. ***** IF YES, CHECK THAT AUDIO RECORDING IS ACTIVATED*** Technical problem? Keep calm!** Explain, apologise and rearrange interview day/date/time

| Are you | | 🞏 male | | | 🞏 female | | | |
| --- | --- | --- | --- | --- | --- | --- | --- | --- |
| How old are you? | | |  |  |  |  |  |  |
|  | 🞏 less than 60 | | 🞏 60 to 64 | | 🞏 65 to 69 | | 🞏 70 to 74 | |
|  | 🞏 75 to 79 | | 🞏 80 to 84 | | 🞏 85 to 89 | | 🞏 90 and over | |
| Who do you live with? | | | |  |  |  |  |  |
|  | 🞏 spouse or partner | | | | 🞏 live alone | | | |
|  |  |  |  |  |  |  |  |  |
|  | 🞏 someone else – please tell us …………………………………………………………………………………………………………………… | | | | | | | |

| Overall, how would you rate your health in the past 4 weeks? | | | | | | |
| --- | --- | --- | --- | --- | --- | --- |
|  | 🞏 excellent | 🞏 very good | 🞏 good | 🞏 fair | 🞏 poor | 🞏 very poor |

**Extract from questionnaire and confirm**

**(Experiences)**

**1. Tell me about how convenient you think your GP practice is for you?**

*What does convenience mean to you?*

*Is it an issue for you? Please tell me more*

*Why do you think this?*

*Could it be more convenient and how?*

**2. Tell me about how convenient you think your pharmacy (chemist) is**

*What does convenience mean to you?*

*Is it an issue for you? Please tell me more. What do you do?*

*Why do you think this?*

*Could it be more convenient and how?*

**3. Please tell me how you usually get your medicines you get on prescription**

*Include ordering the prescription, collecting the prescription, collecting the medicines, how often*

*Is it convenient for you?*

*What does convenience mean to you?*

*Is it an issue for you? Please tell me more. What do you do?*

*Why do you think this?*

*Could it be more convenient and how?*

**4. How does this fit into the things you do every day?**

*Are there any issues and what are they? – tell me more*

*How could this be made easier for you?*

**5. Tell me about when your doctor or anyone else in your GP practice last talked to you about all your medicines you get on prescription to see that they are still working and that you still need them**

*Do you remember who spoke to you and what was discussed?*

*Was this for all medicines or just some of them*

*Do you think your medicines are still working for you and that you still need them?*

*Could your doctor or anyone else in your GP practice do more to help you?*

**5. Tell me about when your pharmacist (chemist) last talked to you about all your medicines you get on prescription to see that they are still working and that you still need them**

*Where did this take place?*

*Do you remember what was discussed?*

*Was this for all medicines or just some of them?*

*Could your pharmacist (chemist) do more to help you with your medicines?*

*Tell me more about this*

*Could your pharmacist (chemist) do more to help you with your health in general?*

*Tell me more about this*

**6. Do you ever speak to your family or friends about your medicines you get on prescription?**

*Tell me about this*

**7. Tell me about how you take your medicines you get on prescription and remembering to take or use them**

*Do you have any issues – tell me?*

*What might help you to make it easier?*

**(Behavioural determinants)**

**Knowledge**

**1. If you needed it, where would you get help or support with getting your medicines you get on prescription?**

*Why would that be your first point for support? Tell me more*

*Have you actually asked for help or support, say in the last 6 months?*

*Did you get the help or support that you needed?*

***2*. If you needed it, where would you get help or support with taking or using your medicines you get on prescription?**

*Why? Tell me more*

*Have you actually asked for help or support, say in the last 6 months?*

*Did you get the help or support that you needed?*

**Social/professional role and identity**

**3. Do you think it is yours or someone else’s responsibility to make sure that you get the medicines that you need?**

*Why? Tell me more*

*If someone else, who and why?*

**Beliefs about Capabilities**

**4. Do you have any problems of difficulties getting your medicines you get on prescription?**

*Tell me more about that - why is it difficult, for how long has this been an issue?*

*What do you do when this happens?*

*What might make it easier for you?*

**5. Do you have any problems of difficulties taking or using your medicines you get on prescription?**

*Tell me more about that - why is it difficult, for how long has this been an issue?*

*What do you do when this happens?*

*What might make it easier for you?*

**Beliefs about Consequences**

**6. How important is it to you to get your medicines you get on prescription?**

*Tell me more about that – why is it so important to you?*

*What would happen if you didn’t?*

*Are any of your medicines more important than others?*

**7. How important is it to you to take or use your medicines you get on prescription?**

*Tell me more about that – why is it so important to you?*

*What would happen if you didn’t?*

*Are any of your medicines more important than others?*

**Memory, Attention and Decision Processes**

**8. How do you remember to get your medicines you get on prescription?**

*Do you have a routine or reminder?*

*Do you ever forget to get your medicines?*

*Tell me about this*

**9. Do you ever choose not to get your medicines you get on prescription?**

*Tell me about this*

*What happens?*

*Do you worry about it?*

**10. How do you remember to take or use your medicines you get on prescription?**

*Do you have a routine or reminder?*

*Do you ever forget to take your medicines?*

*What happens?*

*Do you tell anyone? Who? What do they say?*

**11. A lot of people find it hard to take a lot of medicines. Do you ever choose not to take or use your medicines you get on prescription?**

*Tell me about this*

*What happens?*

*Do you worry about it?*

**Environmental Context and Resources**

**12. Do you have or need any help in getting your medicines you get on prescription?**

*Tell me about this - who, how etc*

*Could anything or anyone else help – tell me?*

**13. Do you have or need any help in taking your medicines you get on prescription?**

*Tell me about this - who, how etc*

*Could anything or anyone else help – tell me?*

**Social Influences**

**14. Do you ever speak to any of your family or friends about getting or taking your medicines you get on prescription?**

*Tell me about this – who, when, what said*

**15. Could any of your family or friends help you more in getting your medicines you get on prescription?**

*Tell me about this – who, how*

**16. Could any of your family or friends help you more in taking your medicines you get on prescription?**

*Tell me about this – who, how*

**Emotion**

**17. Do you ever worry about getting your medicines you get on prescription either now or in the future?**

*Tell me about this – what are you worried about?*

*How you spoken to anyone about this – who, when, what?*

*Is there anything that could be done to help?*

**18. Do you ever worry about taking your medicines you get on prescription either now or in the future?**

*Tell me about this – what are you worried about?*

*How you spoken to anyone about this – who, when, what?*

*Is there anything that could be done to help?*

**Behavioural Regulation**

**19. In the recent past (say the last 6 months), have you changed anything about getting your medicines you get on prescription?**

*Tell me about this – what, why and what happened?*

**20. In the recent past (say the last 6 months), have you changed anything about taking your medicines you get on prescription?**

*Tell me about this – what, why and what happened?*

**Other comments**

**Is there anything else you would like to add about getting or taking your medicines you get on prescription?**
